# Advances in Fluorescent Inorganic–Organic Hybrid Nanostructures: Interfacial and Photophysical Insights for Selective Pesticide Sensing and Removal

**DOI:** 10.3390/nano16171076

**Published:** 2026-08-29

**Authors:** Roberto Acevedo, Harbinder Singh, Mikhael Bechelany, Rajat Bajaj, Jagpreet Singh

**Affiliations:** 1 Facultad de Ingeniería, Universidad San Sebastián, Bellavista 7, Santiago 8420524, Chile; 2 University Centre for Research & Development, Chandigarh University, Gharuan, Mohali 140413, Punjab, India; 3 Institut Européen des Membranes, (IEM), UMR-5635, University of Montpellier, ENSCM, CNRS, Place Eugène Bataillon, CEDEX 5, 34095 Montpellier, France; 4 Department of Mathematics and Natural Sciences, Gulf University for Science and Technology (GUST), Hawally 32093, Kuwait; 5 Centre of Research Impact and Outcome, Chitkara University, Rajpura 140417, Punjab, India; 6 Division of Research and Development, Lovely Professional University, Phagwara 144411, Punjab, India; 7 Bahra Research Innovation & Knowledge Cluster (BRIKC), Rayat Bahra University, Greater Mohali 140103, Punjab, India

**Keywords:** inorganic–organic hybrid nanoparticles, pesticide sensing, fluorescence mechanisms, energy transfer processes, intermolecular interactions, adsorption behavior, catalytic degradation

## Abstract

The extensive use of pesticides in modern agriculture has resulted in their persistent accumulation in environmental systems, posing significant risks to ecosystems and human health. Consequently, the development of integrated strategies for the sensitive detection and efficient removal of pesticide residues has become critically important. In this context, fluorescent inorganic–organic hybrid nanoparticles have emerged as versatile platforms owing to their tunable physicochemical properties and distinctive optical behavior. This review provides a comprehensive overview of recent advances in these hybrid nanomaterials for pesticide sensing and remediation. Particular emphasis is placed on the underlying photophysical mechanisms governing detection, including fluorescence quenching, Förster resonance energy transfer (FRET), inner filter effect (IFE), and photoinduced electron transfer (PET). In parallel, the role of interfacial interactions such as hydrogen bonding, electrostatic attraction, and π–π stacking in adsorption processes is critically discussed. Furthermore, these hybrid systems exhibit high adsorption capacities and rapid removal kinetics, enabling efficient pesticide elimination using both adsorption and catalytic degradation pathways. Overall, this review underscores the potential of fluorescent inorganic–organic hybrid nanoparticles as next-generation materials for sustainable environmental monitoring and remediation of pesticide contaminants.

## 1. Introduction

The ongoing increase in the use of agrochemicals, particularly pesticides, has become essential to meet the growing global food demand. The global market for traditional pesticides and herbicides is projected to reach approximately USD 82.9 billion by 2027, with an expected annual growth rate of 2.7% [1]. However, this extensive use of pesticides has led to severe environmental concerns, particularly water contamination [2]. Several classes of pesticides, including organophosphates, neonicotinoids, carbamates, and pyrethroids, exhibit significant toxicity. With excessive pesticide use, these contaminants can bioaccumulate in environmental matrices and may pose significant risks to ecosystems and human health [3]. Water is essential for all living beings, and more than 700 million people worldwide still lack access to clean drinking water due to the release of toxic contaminants into the environment [4]. Consequently, the demand for clean and safe drinking water is increasing rapidly [5]. Water bodies are continuously being polluted due to chemical exposure from industrial discharge, agricultural runoff, and the accumulation of both organic and inorganic pollutants [2].

Among these, contaminants such as phenols, dyes, heavy metals, detergents, and especially pesticides highly affect the environment and human health [5]. Notably, pesticide residues originating from agricultural activities are considered among the most hazardous pollutants due to their toxicity, persistence, and bioaccumulation potential [6,7]. With rapid urbanization, industrialization, and economic growth, the challenge of providing pollutant-free water is becoming increasingly critical. Therefore, the development of advanced and efficient water treatment technologies is urgently required [7]. Traditionally, HPLC, GC-MS, and other analytical techniques have been widely utilized owing to their high accuracy and sensitivity [8,9]. Nevertheless, these methods are often expensive, time-consuming, and require skilled personnel for handling. Similarly, conventional water treatment approaches, including membrane filtration, reverse osmosis, electrodialysis, chemical oxidation, photodegradation, and adsorption, have been widely employed [10]. However, these techniques often suffer from limitations such as high operational cost, energy consumption, incomplete removal of pesticides, and the generation of secondary toxic by-products. Hence, there is a growing need to develop cost-effective, efficient, and stable treatment strategies with simple operational requirements for the detection and removal of pesticides.

In this context, nanotechnology acts as a boon for environmental monitoring and water remediation. Nanomaterials with dimensions below 100 nm exhibit distinctive physicochemical properties. Among different nanomaterials (NMs), fluorescent NMs are prioritized owing to their rapid response, high sensitivity, and selectivity for real-time monitoring [11,12,13]. Fluorescent-based detection methods rely on mechanisms such as fluorescence quenching, FRET, and IFE, which enable the efficient detection of pesticides. Among the various classes of fluorescent nanomaterials, including semiconductor quantum dots and upconversion nanoparticles, dye-based fluorescent systems and carbon-based nanoparticles, particularly CDs, have attracted significant attention as the most promising candidates for sensing applications. Quantum dots exhibit size-dependent emission, high photostability, tunable fluorescence, and exceptional brightness arising from the quantum confinement effect. In contrast, CDs, also known as carbon quantum dots, represent an alternative class of fluorescent nanomaterials with advantages such as high water solubility, low toxicity, excellent biocompatibility, and ease of surface functionalization. The fluorescence properties of CDs can be easily tuned using heteroatom doping and surface engineering, which enable sensitive and selective detection of target analytes. Furthermore, carbon dots can be readily integrated with organic and inorganic components to form multifunctional hybrid systems. Despite their advantages, bare CDs often suffer from limitations such as low selectivity, reduced quantum yield, and restricted applicability in complex environmental conditions. To overcome these limitations, CDs and other fluorescent materials have been incorporated with a wide range of organic and inorganic components to form hybrid nanoparticles with enhanced performance.

More recently, inorganic–organic hybrid materials have gained a lot of interest for water treatment. These hybrid systems typically consist of an inorganic core, such as metal nanoparticles, quantum dots, or metal oxides, and an organic shell composed of polymers, ligands, or biomass-derived materials [14]. The incorporation of inorganic components, such as metal oxides including titanium dioxide, zinc oxide, and zirconium dioxide, as well as plasmonic nanostructures like gold nanoparticles and silver nanoparticles, is commonly employed to improve charge transfer processes, catalytic activity, and fluorescence modulation [15,16]. These inorganic materials enhance signal transduction in sensing applications and also contribute to photocatalytic degradation pathways for pesticide removal. Similarly, organic components such as functional polymers (e.g., chitosan, polyvinyl alcohol, and polyaniline), supramolecular hosts like beta-cyclodextrin, and biorecognition elements including aptamers are incorporated to improve selective binding and enhance interactions and structural flexibility with pesticide molecules [17,18]. Through the integration of organic and inorganic components, the sensing and adsorption performance can be significantly enhanced via photophysical processes and interfacial interactions. The incorporated organic materials facilitate interactions such as hydrogen bonding, electrostatic attraction, and other surface phenomena, thereby improving sensitivity and selectivity. Furthermore, various mechanisms including PET, IFE, FRET, and quenching are widely employed in sensing applications [19,20]. On the other hand, the removal of pesticides is achieved through adsorption and photocatalytic degradation processes. Adsorption and photocatalytic processes can be used to remove pesticides from water [21]. In the case of adsorption, van der Waals interactions hold pesticide molecules on the surface. However, in the case of photocatalysis, pesticides are converted to less harmful intermediates. Nowadays, organic–inorganic hybrid nanoparticles act as a promising platform for the detection and removal of pesticides. The rational design of such multifunctional hybrid systems provides an effective approach for addressing environmental contamination challenges [15,22,23].

The use of nanomaterials for pesticide sensing has progressed from conventional nanomaterials to multifunctional hybrid systems [24]. In the early stages, researchers utilized metal or other nanoparticles, carbon nanotubes or graphene, and magnetic or quantum dots as recognition or signal transducer platforms [25]. Thus, early studies demonstrated the importance of nanomaterials for both the sensing and the remediation of pesticides. For example, Aragay et al. created a foundation for the use of nanomaterials in pesticide monitoring. This work integrated optical and electrochemical sensors and pesticide degradation and removal by nanomaterials. The authors indicated that nanomaterials have a high surface area, can be modified, and are strong and can bind to specific targets [26].

Later, carbon dots, molecularly imprinted polymers, Metal-Organic Frameworks (MOFs), Covalent Organic Frameworks (COFs), and their hybrid materials were introduced to increase sensitivity and selectivity and extend the versatility of analysis [19,22,27,28]. More recently, research combines sensing, adsorption, and remediation catalysis, especially using sophisticated hybrid architectures. Marimuthu et al. reviewed fluorescent nanomaterial sensors for the detection of pesticides in food and other matrices. Emphasis was given to metal nanoparticles, metal nanoclusters, carbon dots, quantum dots, and their composites. This review described fluorescent sensors based on the mechanisms of Förster resonance energy transfer (FRET), photoinduced electron transfer (PET), inner filter effect (IFE), aggregation-induced emission (AIE), and aggregation-caused quenching (ACQ ) and emphasized the importance of sensitivity, selectivity, portability, and real-time analysis [29].

Although significant contributions have been made, hybrid nanomaterials based on the integration of multiple functionalities, such as fluorescent recognition, adsorption, photocatalytic degradation, and selective interactions, are gaining interest. CDs in combination with metal/metal oxide nanoparticles, MOFs, COFs, and other porous architectures, polymers, and composites are attractive for signal transduction and the removal of pollutants [30,31]. Therefore, this review focuses on recently developed nanomaterials, especially CD-based and hybrid nanomaterial systems for adsorption and remediation of pesticides and their sensing. Emphasis is given to the surface chemistry of the nanomaterials, interaction with interfaces, sensing mechanisms, and the integration of multiple functionalities.

Later, carbon dots, molecularly imprinted polymers, MOFs, COFs, and their hybrid materials were introduced to increase sensitivity and selectivity and extend the versatility of analysis [32]. More recently, research combines sensing, adsorption, and remediation catalysis, especially using sophisticated hybrid architectures.

In this review, we cover the most utilized organic–inorganic hybrid nanoparticles for the detection and removal of pesticides. Then, the detection and removal of pesticides, along with their mechanisms, are discussed. At the end of the study, the application of fluorescent organic–inorganic hybrid nanoparticles in pesticide detection, removal, and future perspectives is also discussed.

## 2. Classification of Pesticides

Pesticides are broadly classified into different categories based on their origin, chemical composition, and target molecules. Pesticides are mainly classified as (i) insecticides; (ii) fungicides; (iii) nematicides; and (iv) herbicides [33]. Based on structure and properties, pesticides are classified into different categories. Organophosphates, organochlorines, carbamates, pyrethrin, and pyrethroids are classes of pesticides [34]. The different classes of pesticides have been shown in a flow chart. Based on their mode of action and chemical structures, pesticides comprise of organophosphates, organochlorines, carbamates, pyrethroids, neonicotinoids, triazines, triazoles, and organophosphates (OPPs). OPP pesticides are a widely known class of pesticides that persist in the environment for longer times. OPPs are phosphorus-containing compounds that act as acetylcholinesterase inhibitors. Triazines serves as herbicides by preventing electron transport during photosynthesis and inhibiting the photosystem. Triazoles mainly function as fungicides by blocking the production of ergosterol, which occurs in fungal cell membranes. Arbamates have processes comparable to those of organophosphates, although they have less persistence and reversible enzyme inhibition. Neonicotinoids target nicotinic acetylcholine receptors, causing overstimulation and paralysis, and pyrethroids, synthetic analogues of natural pyrethrins, interfere with sodium ion channels in insect nervous systems [34]. The statistical analysis revealed that among all pesticides, 50% of pesticides detected in the environment are organophosphates. OPPs can cause pollution, respiratory damage, mutagenesis, carcinogenesis, and even death. Exposure to these toxins cause chronic diseases that significantly lead to death [35]. Significant research has been made in understanding the mechanisms of action and acute effects of pesticides in both humans and animals. There is still a need to perform toxicological research to understand all the consequences. Researchers should focus on the adverse health effects of exposure to pesticides [35].

### Fate of Pesticides

**Figure 1:**
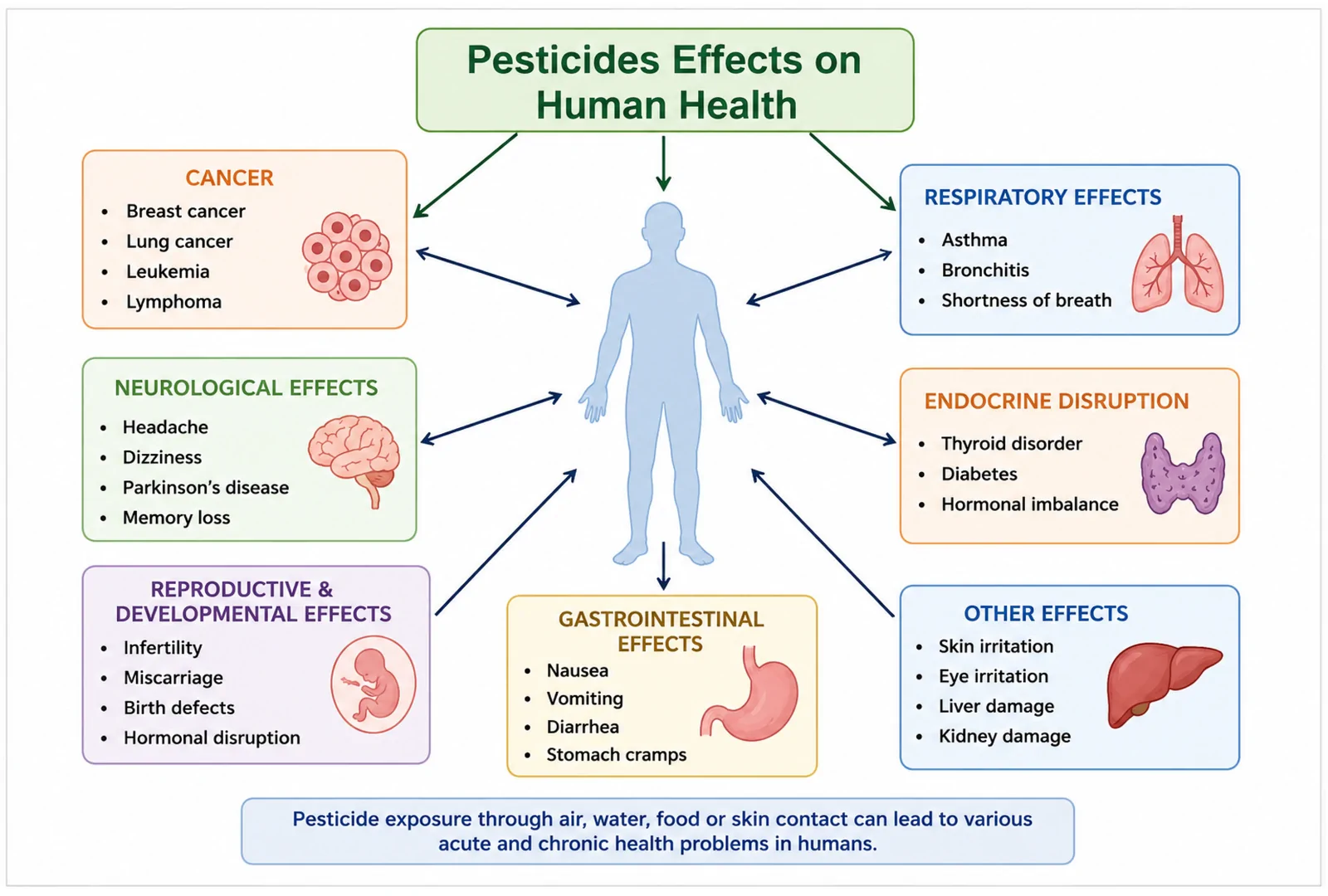
Pesticide effects on human health (original figure drawn by authors).

As per the World Health Organization (WHO), approximately 2.5 million people worldwide face pesticide poisoning. In addition, 0.2 million people die due to pesticide poisoning. More than 80% of pesticides are released from the agriculture sector. The harmful exposure of pesticides predominantly occurs among three groups: (i) industrial workers who are engaged in pesticide manufacturing; (ii) agricultural workers who are engaged in handling and usage of pesticides; and (iii) consumers who are unaware and consuming food contaminated with pesticide residue [35]. The OPP class of pesticides, including chlorpyrifos, malathion, parathion, dimethoate, and dichlorvos has been widely used across the USA and India. Exposure to these harmful contaminants occurs via inhalation, ingestion, or direct contact with skin [36]. The adverse effects of pesticides on human health are shown in Figure 1.

**Figure 2:**
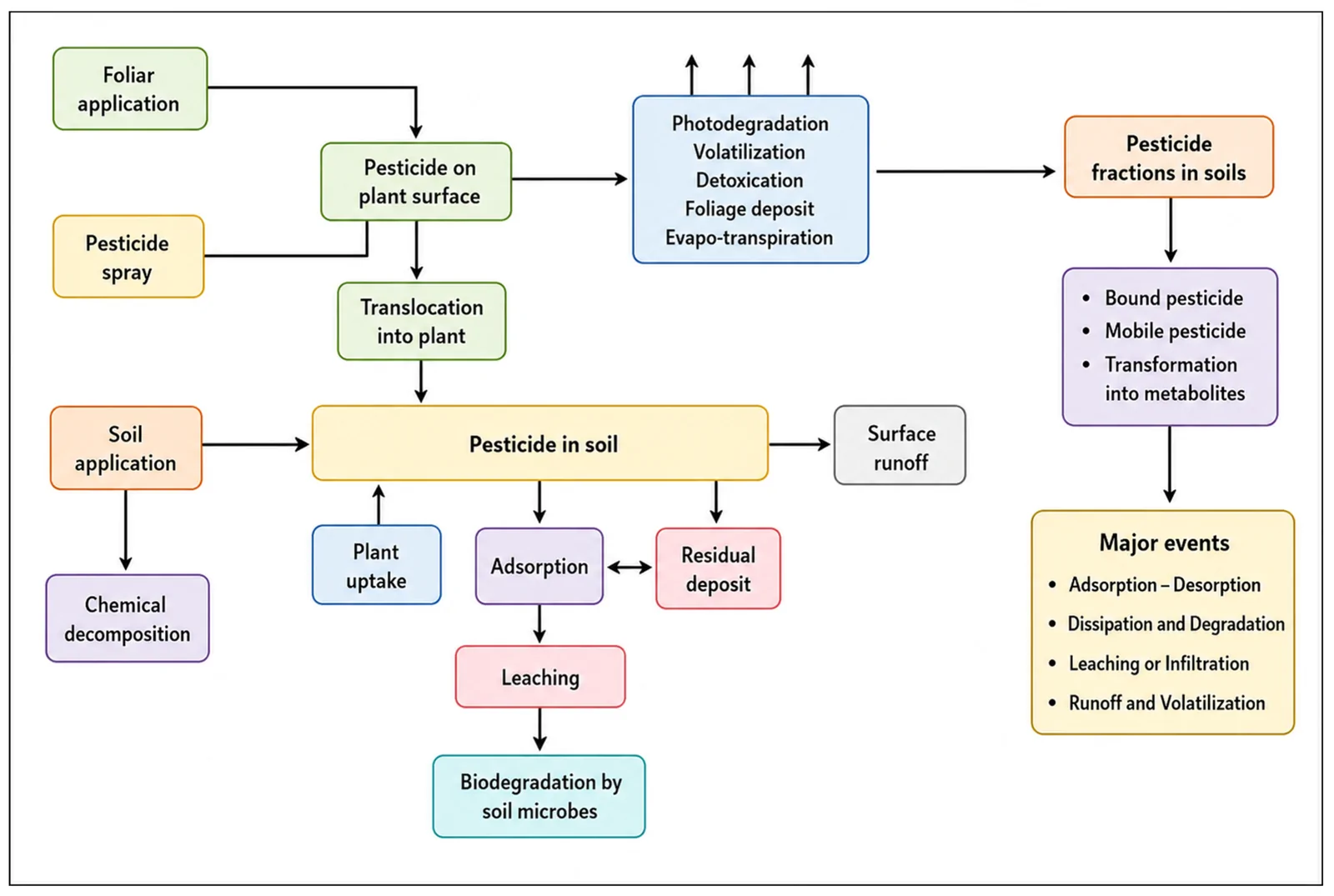
Environmental fate of pesticides showing major transport and transformation pathways (original figure drawn by authors).

Pesticides are used in agricultural fields, and the treatment of vegetables dominates at approximately 90%. In addition, it has been observed that pesticides are continuously used on fruits and vegetables to meet high demand. The increase in the presence of trace amounts of pesticides in food is challenging to human and animal health [37]. Figure 2 shows the environmental fate of pesticides governed by physical, chemical, and biological processes. The pesticide spray in soil interacts with soil components through adsorption and desorption processes. Some pesticides become strongly adsorbed and tend to remain as bound residues, whereas some weakly adsorbed pesticides are more prone to leaching into groundwater. In addition, pesticide residues can be transported to surface water bodies through surface runoff, contributing to environmental contamination.

As per the report by Pesticide Registration and Management Division (PRMD), in Figure 1, the greatest utilization of pesticides among all kinds of pesticides is noted for fungicides, followed by insecticides, herbicides, and rodenticides [37].

## 3. Synthesis Strategies for Hybrid Nanoparticles

The synthesis of fluorescent inorganic–organic hybrid nanoparticles plays a main role in determining their structural, optical, and functional properties. The hybrid nanoparticles are synthesized by incorporating carbon dots, semiconductor quantum dots, or organic dyes with inorganic matrices and organic functional components to achieve enhanced sensing and remediation performance. In such systems, the inorganic part provides stability and catalytic functionality, whereas the organic part provides sensitivity and facilitates specific interactions with target analytes in conjunction with the fluorescent material.

Depending on the fabrication method and type of precursors, these nanohybrids exhibit tunable physical and chemical properties. Various synthesis approaches have been developed and are categorized into bottom-up and top-down approaches. Bottom-up methods involve hydrothermal, co-precipitation, sonochemical, photochemical, and seed-mediated growth techniques. Bottom-up approaches provide control over size, composition, and nanostructure morphology. In contrast, top-down approaches such as laser ablation, atomic beam co-sputtering, and ion implantation involve the modification of bulk materials to produce nanoscale structures with defined characteristics.

### 3.1. Synthesis Approaches (Top-Down and Bottom-Up Approaches)

**Figure 3:**
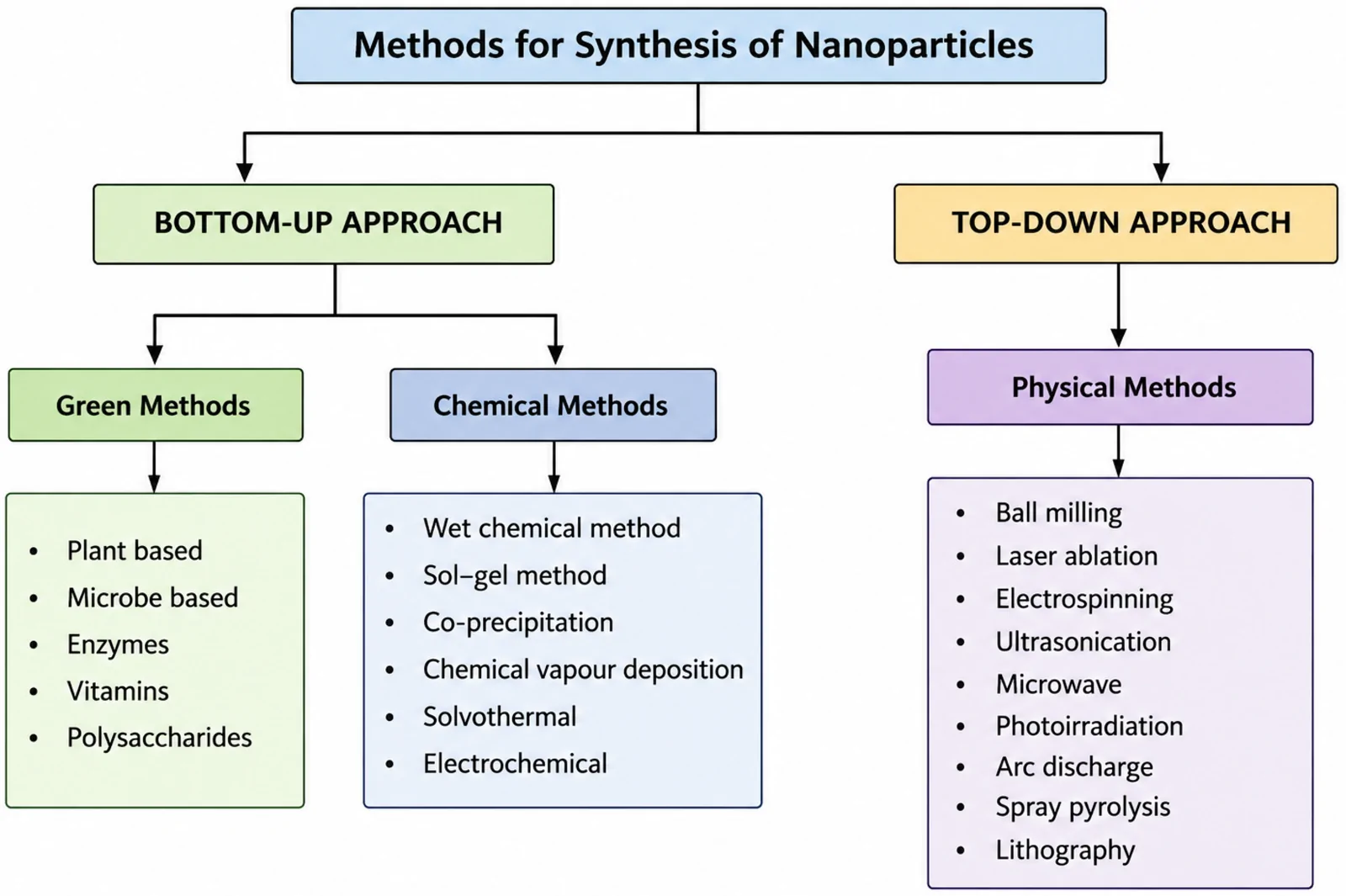
Various methods for the synthesis of nanoparticles (original figure drawn by authors).

Synthesis approaches for hybrid nanoparticles can be broadly divided into bottom-up and top-down strategies as depicted in Figure 3. Bottom-up methods are widely preferred due to their ability to provide precise control over particle size, morphology, and composition, which are essential for achieving desirable optical and functional properties. These methods include hydrothermal, sol–gel, co-precipitation, photochemical, sonochemical, and seed-mediated growth techniques.

On the other hand, physical or chemical breakdown of large materials is used to generate nanoscale structures in top-down approaches. Techniques such as laser ablation, co-sputtering, and ion implantation enable the production of high-purity nanomaterials with controlled surface characteristics. However, these methods are generally less efficient in controlling size distribution compared to bottom-up approaches.

### 3.2. Surface Functionalization and Doping

Surface functionalization and doping play vital roles in tuning the physicochemical and optical properties of fluorescent inorganic–organic hybrid nanoparticles. Heteroatom doping, including nitrogen (N), sulfur (S), and phosphorus (P), is widely employed to modify the electronic structure of fluorescent materials, thereby enhancing fluorescence intensity and tuning emission properties [38].

In addition, ligand modification is used to improve nanoparticle stability, dispersibility, and selectivity by introducing functional groups that facilitate specific interactions with pesticide molecules. Advanced strategies such as molecular imprinting and ionic liquid functionalization further enhance selectivity and sensitivity by creating tailored binding sites and favorable microenvironments for analyte interaction. Various studies have reported the synthesis of NCs using different modifications. For instance, Ali et al. synthesized fluorescent magnetic molecularly imprinted polymer ( FMMIPs), and magnetic non imprinted polymers (MNIPs )using a free radical polymerization technique [39]. Wang et al. prepared CD@MIP using the SI-ATRP method [40]. In another study, Wang et al. developed PCN-224@CDs for the detection of malathion, phoxim, chlorpyrifos, and omethoate, [41]. In addition, Liang et al. developed nitrogen doped carbon dots ( N-CQDs) with a hydrothermal method using corn starch as a natural precursor for the selective detection of Fe^3+^/glyphosate [42].

## 4. Detection of Pesticides Using Hybrid Nanoparticles

The development of fluorescent inorganic–organic hybrid nanoparticles for the detection of trace amounts of pesticide residues has turned out to be a highly selective and sensitive method. This kind of hybrid NP combines the unique fluorescence of nanostructures with the ability of inorganic and organic surfaces to interact selectively with target molecules. Detection of analytes with the help of these particles takes place mainly through photophysical processes. Heteroatoms and chemical groups can further improve interaction with the analyte and thus increase the efficiency of the detection procedure.

From an analytical perspective, understanding the direction of the fluorescence response is crucial. Fluorescence turn-off (ON–OFF) sensors are always employed because the quenching of pesticide-induced fluorescence can be easily correlated to the amount present. However, it should be noted that fluorescence may be attenuated by quenchers or changes in the environment. On the other hand, it is straightforward to interpret a positive response in the case of turn-on (OFF–ON) sensors. Therefore, in order to achieve highly selective and reliable sensors for pesticide detection, an increased effort is warranted for the development of turn-on and ratiometric fluorescence techniques.

In recent years, several studies on hybrid nanomaterials as effective sensors for detecting pesticides, such as organophosphates, carbamates, and fungicides, were carried out. The most commonly used modes include “turn-on,” “turn-off,” and ratiometric fluorescence-sensing methods. In some cases, the use of a dual-emission system and multiple detection channels allows increasing reliability and accuracy. This section mainly focuses on recent advances concerning the application of fluorescent inorganic–organic hybrid nanoparticles as sensors for detecting pesticides. Table 1 shows a summary of fluorescent organic–inorganic hybrid NMs for the detection of pesticides.

Li et al. synthesized N, S, B, and flavonoid co-doped CDs derived from the ginkgo biloba leaves for the detection of pesticides such as fenitrothion (Fen), dithianon (Dit), and dinoseb (Din). The incorporation of heteroatoms and a flavonoid moiety contributes to improved fluorescence properties and sensing performance. The sensing mechanism was attributed to the spectral overlap between the UV–vis absorption of the pesticides and the excitation/emission spectra of the carbon dots, leading to an inner filter effect (IFE) and Förster resonance energy transfer (FRET), along with non-covalent interactions facilitating energy transfer processes [38].

Man et al. developed heteroatom-doped CDs to enhance the detection of OPPs. The authors utilized stems and leaves of broccoli for the preparation of CDs using a hydrothermal method. Furthermore, the system CDs/Fe^2+^/acetylcholinesterase (AChE) was developed to detect OPP residues in real food samples. The excitation-dependent fluorescence CDs exhibits high stability at different experimental parameters with good fluorescent properties. The prepared CDs show multicolor fluorescence due to large numbers of functional groups present on its surface. Furthermore, to ensure the stability of CDs, the pH and ionic strength of PL intensity were further investigated. The prepared CDs exhibit high water solubility, stability, and luminescence properties at different pH values and ionic strengths. The hydroxyl and amine groups of CDs can easily coordinate with Fe^3+^ ions to allow photoinduced excitation [43].

Chen et al. developed a ratiometric fluorescent sensor based on N-CQDs and copper nanoclusters for the detection of thiram and paraquat. The detection mechanism is governed by a combination of fluorescence resonance energy transfer (FRET) and analyte-induced fluorescence modulation. N-CQDs act as the energy donor, whereas CuNCs serve as the acceptor, resulting in FRET process due to the spectral overlap that establishes a stable dual-emission system. The introduction of target pesticides forms no new complex, and there is no change in fluorescence lifetime. This indicates that FRET is not the dominant mechanism. Rather, electron transport and electrostatic interactions are the main mechanisms that cause fluorescence modulation. In particular, the negatively charged N-CQDs@CuNC system interacts with positively charged paraquat, promoting electron transport and resulting in fluorescence quenching. When thiram interacts with copper species, disulfide bonds are broken, and Cu–thiram complexes are formed, both of which aid in the quenching of fluorescence. Analyte-specific interactions are thus layered on a FRET-based ratiometric platform to provide the sensing response [44].

Fu et al. developed an “on–off–on” fluorescent sensor for sensitive detection of thiram. The nitrogen doped carbon dots (N-CD) surface contains hydrophilic functional groups, including carboxyl, hydroxyl, and amine groups, which enhance its water dispersibility. The functional groups (carboxyl, hydroxyl, and amine) present on the CD surface exhibit high selectivity toward the thiram molecules. The negative zeta potential of N-CDs was negative owing to the presence of citrate ions on its surface. Electrostatic repulsion between the negatively charged N-CDs and AuNPs contributes to their separation. The fluorescence quenching on N-CDs was attributed to the IFE or FRET between them. The CDs exhibit blue fluorescence, which follows an on–off–on pattern of N-CD fluorescence. Moreover, IFE was identified as the dominant quenching mechanism. To avoid interference in complex systems, IFE-based methods should be improved for better accuracy and reliability [45].

The detection of pesticides including thiram, chlorpyrifos, and carbaryl has been done by Wang et al. using a CDs/SiO_2_ probe [44,45]. The prepared CDs show dual signals under excitation at various wavelengths. The authors incorporated SiO_2_ NPs in carbon dots prepared by pomegranate seeds, L-aspartic acid, and L-tryptophan (PAT-CDs), resulting in the emission of blue fluorescence. The phosphorescence emission of PAT-CDs@SiO_2_ contributes significantly to the observed optical response. Moreover, the long phosphorescence lifetime was easily detectable by the naked eye. The SiO_2_ shell acts as protective isolation from the phosphorescence quencher in aqueous solution for multi-channel detections. The thiram–Cu(II) complex quenches both the fluorescence and phosphorescence of PAT-CDs@SiO_2_ through IFE [46].

Further, the study revealed that the pH of PAT-CDs-SiO_2_ could influence its fluorescence intensity. The study reported that fluorescence intensity increases as pH increases from 3 to 11. On the other hand, phosphorescence remains unaffected by varying the pH from 3 to 11.

Fluorescent CDs for the detection of Fe^3+^ and glyphosate have been synthesized by Liang et al. using a hydrothermal approach. The nitrogen-doped CQDs were prepared with a hydrothermal method using precursors like dialdehyde starch and 1,8-diaminonaphthalene. The optical characteristics of CQDs were influenced by surface functional groups. The surface of N-CQDs was rich with hydroxyl, amine, and carbonyl groups. Mechanistically, fluorescence switching arises from the coordination between surface functional groups and Fe^3+^ ions, producing a chelation-induced fluorescence enhancement. The resulting N-CQDs exhibit blue–green fluorescence, whereas the corresponding bare CDs lack a fluorescence emission band around 425 nm. Glyphosate coordination in fluorescence quenching can further combine the conjugation between N-CQDs and Fe^3+^ ions. Glyphosate’s excellent coordination ability allows it to readily form stable intermediates with metal ions [42].

Yang et al. developed a ratiometric fluorescence probe using a hydrothermal method for the detection of pesticide thiabendazole. The as-prepared CDs show dual emission with high fluorescence stability. Due to the inner filter effect, thiabendazole (TBZ) exhibits turn-off fluorescence. The probe provided satisfactory recoveries for TBZ determination in real fruit juices. The IFE was identified as the primary mechanism responsible for fluorescence quenching. The increase in fluorescence intensity upon TBZ addition further supports the contribution of both dynamic and static quenching processes. In DQE, the excitation state fluorophore is non-radiometrically deactivated upon collision with quencher. Furthermore, the formation of a ground-state complex between the fluorophore and quencher shows the occurrence of SQE [47].

Alhazzani et al. developed a copper dimethyldithiocarbamate-incorporated CD system for ziram detection. The detection of ziram follows coordination interactions and a photophysical quenching process using blue and red emission carbon dots(B/NSCDs, R/NSCDs). Upon Cu^2+^ addition, a complex forms through coordination of Cu^2+^ with negatively charged surface functional groups. The high binding affinity of dithiocarbamate triggered the ligand displacement for Cu^2+^. The formation of the stable complex copper dimethyldithiocarbamate (CuDDC)_2_ emits a yellow color. IFE appears to be the dominant quenching mechanism, with an additional contribution from static quenching. Moreover, static quenching plays a role in this sensing process. This interpretation is supported by the Stern–Volmer analysis and is consistent with the formation of non-fluorescent ground-state complexes between the sensing compound and ziram molecules. The detection approach utilized a combined effect in which quenching is achieved through complexation and ligand displacement of the Cu^2+^ ion by the target molecule. The reported quantum yield was 12% for R/NCDs and 16% for B/NSCDs [48].

Alhazzani et al. further evaluated the effect of pH on fluorescence intensity over the range of 2–10, where the fluorescence intensity increased with pH and reached a maximum near pH 7. The maximum fluorescence intensity was reached at around pH 7. Beyond this, the pH further decreases, which happens due to the occurrence of protonation or deprotonation in functional groups. The fluorescence intensity decreases at high pH values due to hydroxide-induced interference and reduced stability of Cu^2+^ complexes [48].

The ionic strength affects the stability of fluorescence, which was investigated by varying the concentration of NaCl. The increase in ionic strength enhances the photoluminescence intensity of CDs, defining their resilience in solutions of differing ionic strengths.

Wang et al. developed a CD-incorporated molecularly imprinted polymer (CD@MIP) for selective detection of MCPA. The sensor showed good linearity over 0–80 μmol/L and efficient fluorescence quenching. The quenching mechanism was mainly attributed to the electron charge transfer interactions and Meisenheimer complex formation. The electron transfer occurs between the electron-deficient amino groups on the surface of CDs and electron-rich MCPA. This interaction facilitates photoinduced electron transfer, while hydrogen bonding further stabilizes the complex, ultimately leading to fluorescence quenching [40].

Tang et al., developed multicolor nitrogen dots for the efficient detection of thiram and chlorpyrifos. M-Ndots were synthesized by microwave irradiation. In this study, Cu^2+^ and Fe^3+^ initially quench the fluorescence of the carbon dots, producing an “off” state. For thiram, degradation products react with Cu^2+^, removing the quencher and restoring fluorescence (“on” state). However, chlorpyrifos blocks the activity of acetylcholinesterase and consequently prevents the oxidation of Fe^2+^ to Fe^3+^, thus avoiding fluorescence quenching [49].

Arroyave et al. reported a CD- and graphene oxide-based fluorescent aptasensor for the detection of chlorpyrifos. The sensor operates through a FRET-based turn-off/turn-on mechanism. The mechanism involves the quenching of CDs upon interaction with graphene oxide due to strong π–π stacking with the aptamer. Binding of chlorpyrifos induces structural rearrangement of the aptamer, causing its detachment from GO and restoring fluorescence. Moreover, the molecular docking exhibits stem-loop structures, and guanine-rich regions of the aptamer play a key role in stabilizing the aptamer–chlorpyrifos complex [50].

Wang et al. reported the synthesis of a thioctic-derived CD-based fluorescent sensor for the detection of thiophanate-methyl based on a turn-off/turn-on mechanism. Initially, fluorescence was quenched; subsequent addition of thiophanate-methyl led to formation of stable TM–Hg complexes. The formation of complexes suppresses electron transfer interactions responsible for fluorescence quenching, thereby resulting in fluorescence recovery. Fluorescence recovery results from suppression of non-radiative decay, allowing excited electrons to return to the ground state through radiative pathways. The absence of a noticeable change in fluorescence lifetime supports the formation of non-fluorescent ground state complexes and therefore a predominantly static quenching mechanism. Overall, the interaction between TM–Hg complexation-driven fluorescence recovery and electron transfer-induced quenching controls the sensing response [51].

Han et al. synthesized nano-hybrid CDs for the efficient detection of carbendazim in fruit and vegetable samples. The study reported the lanthanide-hybridized ratiometric sensor PAN-CDs&DPA-Tb. Detection is governed by π–π stacking interactions between the aromatic ring of carbendazim and the benzene moieties of the CD composite. These interactions promoted photoinduced charge transfer from electron donating groups of carbendazim to electron-accepting functional groups, leading to fluorescence quenching. Furthermore, the analyte–sensor hybrid is further stabilized by hydrogen bonding interactions, which improve electron transfer efficiency. In addition to PET, the quenching process is supported by the inner filter effect (IFE), in which carbendazim absorbs certain amounts of the excitation/emission light, reducing the measured fluorescence intensity. For carbendazim detection in complicated matrices, the ratiometric dual-emission approach enhances selectivity and reliability, while PET and IFE perform in conjunction to increase sensitivity [52].

Yu et al. reported a ratiometric sensor based on carbon dots hybridized with Cu-based metal–organic frameworks (CDs@Cu-MOFs) for detection of thiophanate-methyl. The sensing response involves π–π stacking, metal–ligand coordination, and modulation of electron transfer pathways. The CDs coordinate with the Cu-MOF through interactions between Cu^2+^ ions and surface carboxyl groups. The incorporation of thiophanate-methyl exhibits affinity toward Cu ions, leading to the formation of a stable TM–Cu complex. The Cu-MOF framework structure is disrupted by this competitive coordination, which releases carbon dots and organic ligands into the solution. Fluorescence intensity is greatly increased at both emission wavelengths as a result of the suppression of the electron transfer and LMCT pathways. Thus, framework disruption, suppression of electron transfer, and complex formation collectively generate the ratiometric response (dual emission), which allows for sensitive thiophanate-methyl detection [53].

Wang et al. developed a ratiometric fluorescence sensor for organophosphorus pesticides, including malathion, phoxim, chlorpyrifos, and omethoate. The probe consisted of CDs integrated within zirconium-based MOFs (PCN-224@CDs). PCN-224@CDs have been synthesized using solvothermal synthesis followed by in situ assembly for the detection of OPPs. Detection involves spectral overlap between CD emission and PCN-224 absorption, facilitating FRET, together with coordination between Zr centers and pesticide phosphate groups. In particular, coordination interactions among Zr active sites and pesticide phosphate groups, as well as Förster resonance energy transfer (FRET) between carbon dots and the MOF framework, governed the sensing mechanism. Electron transfer processes and spectral overlap-driven fluorescence modulation provided additional support [41].

Kumar et al. reported the detection of chlorpyrifos using metal-free fluorescent sensing based on pyrene-functionalized organic cationic receptor nanoparticles (R1-ONPs). The turn-on response originates from excimer formation. NMR spectral shifts indicate that the receptor (such as –NH and imine groups) and the pesticide molecule form strong hydrogen bonds upon engagement with chlorpyrifos. A tighter stacking of aromatic units results from the simultaneous promotion of π–π interactions between pyrene moieties. This structural change makes it easier for pyrene excimers to form, which leads to the appearance of a new emission band and a notable increase in fluorescence. The stabilization of the excited state upon complex formation is further supported by the increase in fluorescence lifetime. In general, synergistic hydrogen bonding and π–π stacking interactions that promote excimer production and increase fluorescence intensity are responsible for the sensing response [54].

When assessing the analytical reliability of pesticide sensors, the reliability of the fluorescence response is of particular importance. For most cases, sensors based on fluorescence turn-on exhibited lower background responses, while simpler sensors based on fluorescence turn-off may be subject to ambiguity caused by nonspecific quenching. This is especially true for transition metal ion sensors. For instance, in a fluorescence turn-on system, the paramagnetic Cu^2+^ quenches the fluorescence by providing an excitation pathway. Therefore, the quenching of a fluorescent system in the presence of Cu^2+^ should be interpreted with caution since it can be attributed most likely to the paramagnetic nature of Cu^2+^ rather than to the recognition of a particular pesticide. Therefore, turn-on and ratiometric systems, as long as they are based on relevant interferent controls and a sufficient level of evidence, can give better selectivity in the detection of pesticides.

**Figure 4:**
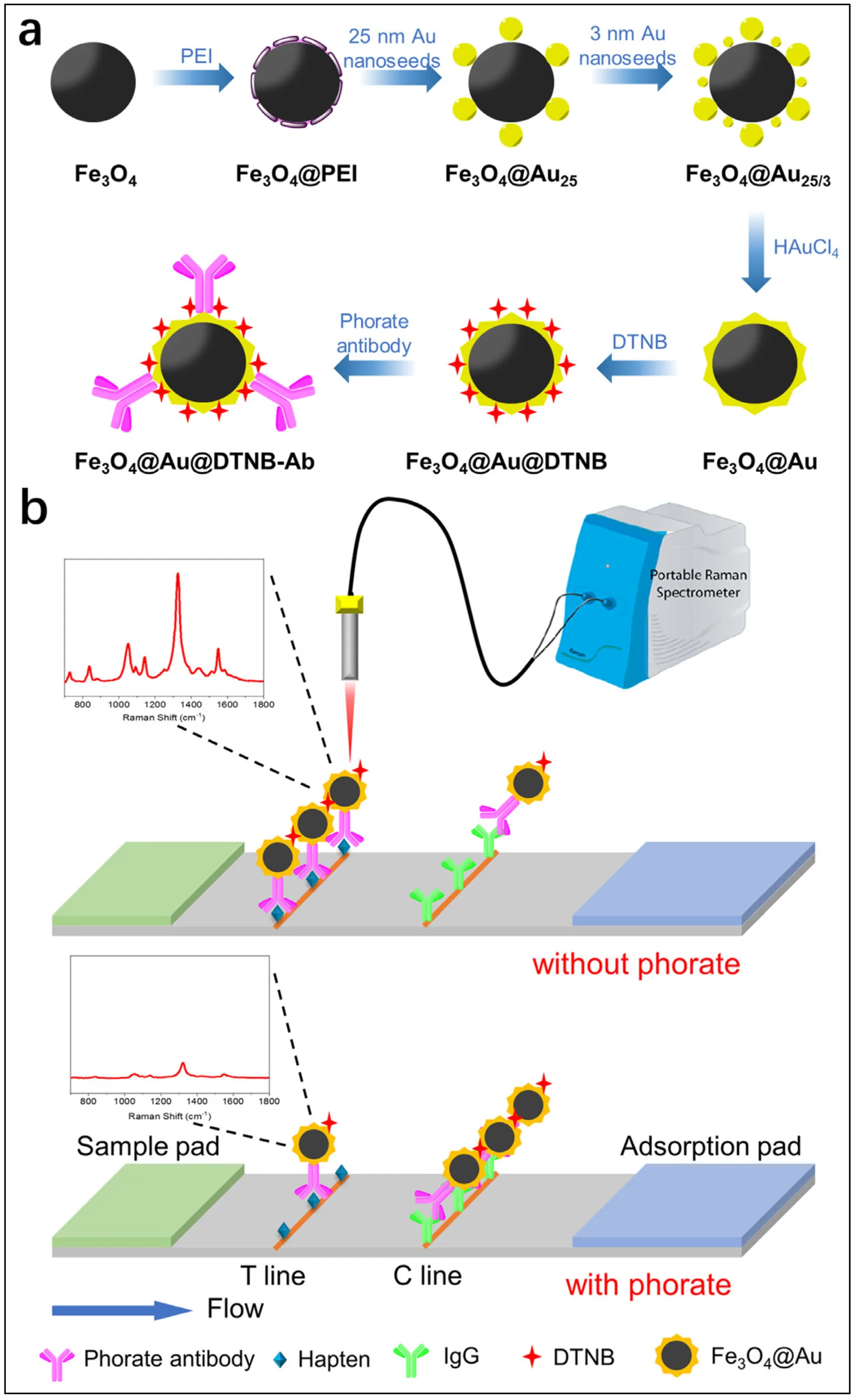
Schematic shows the (**a**) preparation process and (**b**) sensing mechanism of a NP-based sensing probe (reproduced from ref. [55] under the Creative Commons CC-BY license).

The reported selectivity of CD-based pesticide sensors is typically established by challenging the sensing platform with a mixture containing select metal ions, inorganic ions, pesticides, amino acids, carbohydrates, organic acids, and other matrix constituents. The studies have employed different mixtures of interfering agents, making comparison of selectivity reports practically impossible. For example, the CDs@Cu-MOF probe for thiophanate-methyl was reportedly tested against K^+^, Na^+^, Mg^2+^, Zn^2+^, glucose, sucrose, fructose, malic acid, tartaric acid, oxalic acid, arginine, and other potential interferents, while the R1-ONP system was challenged against competing pesticides and common interfering anions. Similar to these examples, the PAN-CDs &DPA-Tb system also showed a differential response to carbendazim in the presence of other co-existing materials. In lieu of a comparison of selectivity of different sensing systems, the analytical performance of each sensor should be judged based on the nature and concentration of the interfering materials relative to the target pesticide [54]. Figure 4 shows the development of a hybrid NP-based sensing probe for the effective detection of phorate through a SERS mechanism [55].

**Table 1:** Summary of fluorescent organic–inorganic hybrid NMs for the detection of pesticides highlighting synthesis strategies, particle size, sensing mechanisms (IFE, FRET, PET, π–π interactions, etc.), performance parameters, and their applicability in real samples.

No.	Material	Pesticide	Detection Mode	Sensing Response	Mechanism	Linear Range	LOD	LOD Determination Method	Selectivity/Interferents Tested	Recovery (%)	Real Sample	Ref.
1	AuNPs/N-CDs	Thiram	Fluorescence	ON–OFF	IFE	10–200 ng/mL	4.7 ng/mL	NR	NR—verify against original selectivity experiment	102.22–107.57	Hawthorn	[45]
2	N-CQDs@CuNCs	Thiram; paraquat	RF	ON–OFF/ratio change	FRET	Thiram: 10–500 nM; paraquat: 5–100 nM	7.49 nM; 3.03 nM	NR	NR	82–114; 70–120	Nelumbinis semen, Coix lacryma-jobi, ginseng	[44]
3	N-CDs@PCN-222	Glyphosate	RF + colorimetry	OFF–ON/ratio change	PET	0.01–6.67 mg/L	9.06 μg/L	NR	Multiple pesticide/metal-ion interferents; exact list after checking SI	94.0–112.5; 90.0–113.6	Rice, tap water	[56]
4	ssDNA aptamer-CDs-GO	Chlorpyrifos	Fluorescence	ON–OFF	π-stacking/electrostatic interactions	NR	0.01 μg/L	NR	Aptamer-based selectivity; exact interferents from original study	84.3; 125.13	Santa Elena; San Cristóbal	[50]
5	B/NSCDs@Cu + R/NCDs	Ziram	RF	ON–OFF/ratio change	IFE, FRET	0.02–1.0 μg/mL	0.006 μg/mL	NR	NR; exact tested pesticides/ions should be verified	98.75–101.00	Apple, wheat grain, river water	[48]
6	N-CQDs	Fe^3+^; glyphosate	Fluorescence	ON–OFF	Aggregation-induced quenching	Fe^3+^: 0.1–36 μmol/L; glyphosate: 0–45 μmol/L	0.024; 0.036 μmol/L	NR	NR	NR	NR	[42]
7	PAT-CDs@SiO_2_	Carbaryl; thiram; chlorpyrifos	Dual fluorescence/phosphorescence	ON–OFF	IFE, FRET	FL: 0.1–1.0 μg/mL; PL: 0.025–1.0 μg/mL	FL LOD = 45.8 ng/mL; PL LOD = 16.3 ng/mL	NR	Multiple pesticides; exact interferents should be stated	90–110	Tap water, pork	[46]
8	Carbon dots	Thiabendazole	RF	ON–OFF/ratio change	IFE, SQE/DQE	0–1000 μmol/L	0.15 μM	NR	NR	96.73–111.17	Pear juice	[47]
9	N-CD-Fla	Fenitrothion; dithianon; dinoseb	Chemosensor/fluorescence	ON–OFF	IFE, FRET	Fen: 1–2000 nM; Dit: 1–1000 nM; Din: 1–3000 nM	0.36; 0.28; 0.66 nM	NR	Multiple pesticide interferents; exact list should be added	91.0–102.3	Rice	[38]
10	N-, S-, Si-doped CDs	Organophosphorus pesticides	Fluorescence	ON–OFF	AChE inhibition	0–200 μM	1.43 μM	NR	OPP selectivity/interference test should be specified	90–101	Food samples	[43]
11	CD@MIP	MCPA	Fluorescence	ON–OFF	Meisenheimer complex formation/PET quenching	0–80 μmol/L	3.5 μmol/L	3σ method	Molecular imprinting provides MCPA recognition; exact competing analytes should be stated	90.5–98	Cabbage	[40]
12	M-Ndots	Thiram; chlorpyrifos	Fluorescence	ON–OFF	Gas membrane separation/derivatization	0.5–2.5; 0.01–0.50 μg/mL	0.1; 0.002 μg/mL	NR	NR	90–115	Fruits, vegetables	[49]
13	SCDs	Thiophanate-methyl	Fluorescence	OFF–ON (after Hg^2+^-induced OFF state)	Static quenching/S–Hg affinity	0.05–5.0 μmol/L	0.0076 μmol/L	3σ/KSV, n = 3	Other common metal ions and pesticides	96.28–110.23	Tap water, grape juice, CRP	[51]
14	PAN-CDs&DPA-Tb	Carbendazim	RF	ON–OFF	PET, IFE, π–π stacking	0–0.091 mmol/L	3.0 × 10^−3^ μmol/L	3σ/S	Coexisting substances tested; only MBC produced significant quenching	92.22–106.08	Pear, cucumber, spinach	[52]
15	CDs@Cu-MOFs	Thiophanate-methyl	RF	OFF–ON	π-stacking/Cu^2+^ coordination	0.0307–0.769 μmol/L	3.67 nmol/L	3σ rule	K^+^, Na^+^, Mg^2+^, Zn^2+^, glucose, sucrose, fructose, malic acid, tartaric acid, oxalic acid, Arg, and pesticides	93.1–113	Food samples	[53]
16	PCN-224@CDs	Malathion; phoxim; chlorpyrifos; omethoate	RF	ON–OFF/ratio change	FRET	0–1500 ng/mL	4.57; 2.69; 5.88; 7.94 ng/mL	S/N = 3	Nine pesticides tested	75.51–115.74	Celery, leek	[41]
17	CDs/DTNB	Chlorpyrifos	Fluorometric/colorimetric	OFF–ON	IFE/AChE inhibition	0.3–200 ng/mL	FL LOD = 0.081 ng/mL; colorimetric LOD = 0.054 ng/mL	NR	OP interference/selectivity tested	93.3–108.8	Tap water, pork	[57]
18	R1-ONPs	Chlorpyrifos	Fluorescence	OFF–ON (turn-on)	Pyrene-excimer formation/PET suppression	0–120 μM	18.9 nM	3σ method	Competitive pesticides + common interfering anions	~95	Food, water, soil	[54]

## 5. Removal of Pesticides Using Hybrid Nanoparticles

Nanomaterials are promising for pesticide adsorptive removal and catalytic transformations [22]. Adsorptive removal systems “capture” pesticide molecules by surface interactions, whereas catalytic transformation systems convert pesticide molecules to less harmful species. Both processes may be enhanced by the large surface area, porosity, and surface reactivity of nanomaterials. Pesticide adsorption and reactions may be optimized by nanomaterials functionalized with proper catalytic sites and/or adsorption sites. The early work of Aragay et al. demonstrated several potential uses of nanomaterials for the removal and degradation of pesticides and contributed significantly to the following developments of advanced multifunctional hybrid systems [26].

Pesticide removal generally occurs through adsorption, photocatalytic degradation, or a combination of both processes. Adsorption-based removal involves a number of interfacial interactions, including as π–π stacking, H-bonding, electrostatic attraction, van der Waals forces, and coordination interactions between the adsorbent surface and pesticide molecules. Furthermore, advanced hybrid systems frequently display photocatalytic activity, in which pesticide molecules undergo breakdown down into less hazardous compounds by reactive oxygen species (ROS). The interaction strength and adsorption efficiency are further improved by the inclusion of functional groups, heteroatoms, and active metal sites.

In order understand the nature of adsorption processes, isotherm and kinetic models, such as Langmuir and Freundlich isotherms, as well as pseudo-first-order and second-order kinetic models, are most employed to evaluate adsorption behavior. Several studies reported the adsorption of pesticides using hybrid NPs. Importantly, some multifunctional hybrid materials integrate pesticide detection and adsorption/removal systems in one single platform. One system responds optically to pesticides in a measurable way and at the same time captures and removes the pesticides. An integrated system eliminates the need for discrete monitoring and remediation processes. Some examples of these systems are CD@MIP for MCPA, kgd-M1@ACPs for DCN, and Fe_3_O_4_@SiO_2_@UiO-67 for glyphosate. Of these, Fe_3_O_4_@SiO_2_@UiO-67 was synthesized as a smart MOF-based adsorbent for the selective, fluorescence detection and removal of glyphosate. This system provides a basis for the integration of sensing and remediation into a single material. Table 2 shows the detection and adsorption performance of representative hybrid nanomaterials for pesticide monitoring and removal.

### 5.1. MOF-Based and MOF Hybrid Materials

Yang et al. designed a pH-sensitive smart adsorbent for the selective removal of glyphosate based on magnetic, fluorescent, and MOF materials. This adsorbent integrates Fe_3_O_4_, SiO_2_, and UiO-67. The phosphate of glyphosate is attracted to the Zr–OH groups of UiO-67. The magnetic Fe_3_O_4_ allows for simple separation of the adsorbent using an external magnetic field. The adsorbent gave a linear fluorescence response in the range of 0.1–40 mg L^−1^, and the detection limit of glyphosate was found to be 0.093 mg L^−1^. The adsorbent showed a maximum adsorption capacity of 256.54 mg g^−1^, and adsorption completed in 60 min following a pseudo-second-order kinetics model. The Langmuir model provided a more favorable fit, indicating interaction with active sites that be more or less similar. XPS data also confirmed the formation of Zr–O–P bonds. More importantly, there was no significant decrease in adsorption after the fourth cycle, demonstrating the reusability of the adsorbent. Therefore, this magnetic fluorescent UiO-67 adsorbent integrated multifunctional detection and adsorption/removal [58].

Zhu et al. developed Zr-based MOFs of UiO-67 for the efficient removal of pesticides glyphosate (GP) and glufosinate (GF). The adsorption capacity reached 537 mg g^−1^ for GP and 360 mg g^−1^ for GF. Adsorption increased rapidly during the first 60 min and reached equilibrium after 150 min for GP and 200 min for GF. This sudden increase in adsorption rate in the first 60 min was due to vacant adsorption sites. After a certain time period, the adsorption rate starts decreasing due to blocked cavities. The adsorptive kinetics suggest the study follows a pseudo-second order reaction, which forms due to the chemisorption process involving valency forces via exchange of electrons between the adsorbate and the adsorbent. The chemical interactions occur between phosphate groups of pesticides and Zr–OH functional groups. Zr_6_O_4_(OH)_4_ clusters act as active binding sites, facilitating the formation of stable Zr–O–P coordination bonds. Additionally, the UiO-67 structure produces additional reactive Zr-OH sites to enhance the adsorption capacity. Thus, coordination-driven binding is the dominant mechanism for OPP removal [59].

**Figure 5:**
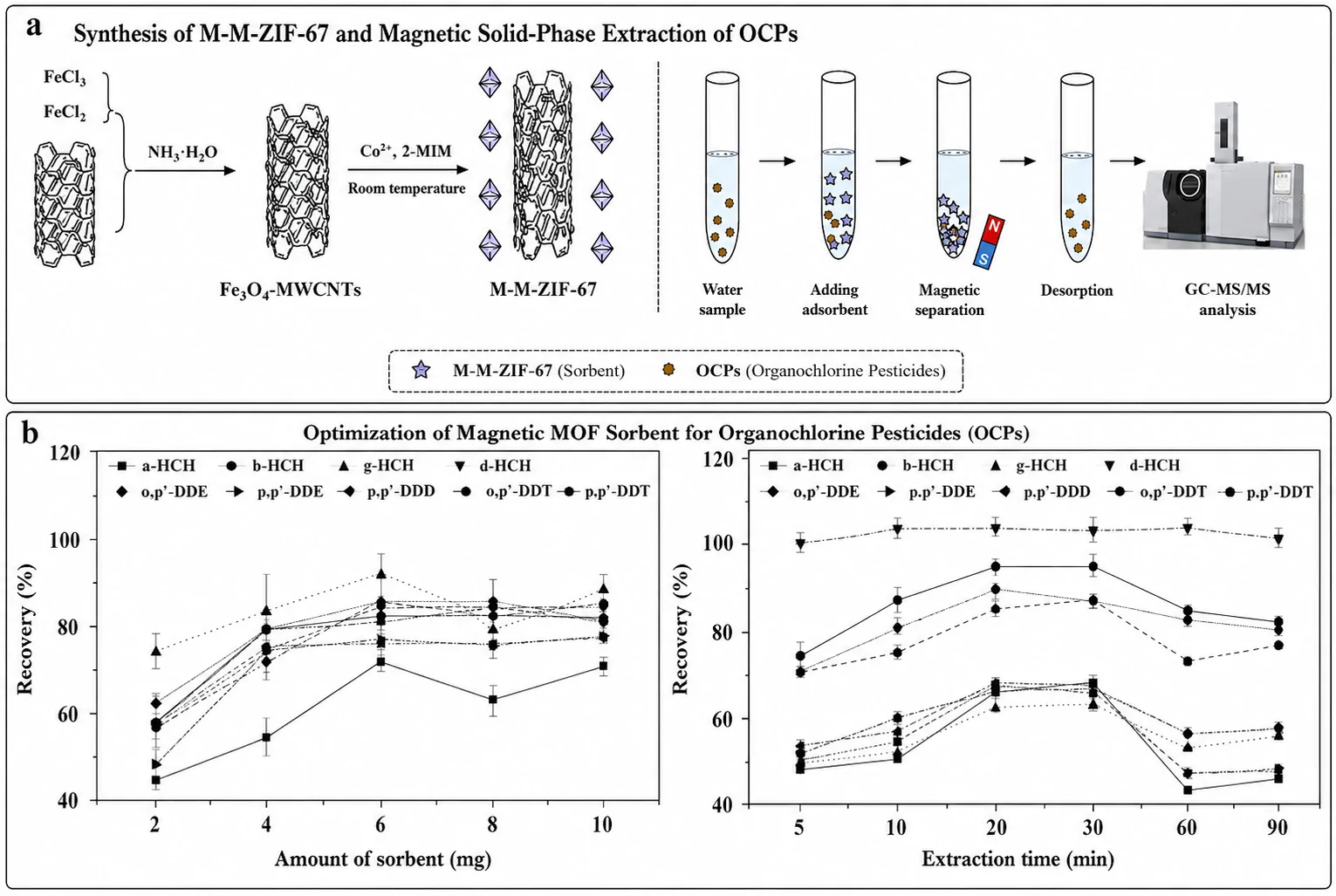
Schematic shows the (**a**) synthesis of zeolitic imidazolate frameworks and (**b**) adsorption studies against various pesticides (reproduced from ref. [61] under the Creative Commons CC-BY license).

Jia et al. developed portable MOF–alginate composite beads (kgd-M1@ACPs) for the sensitive detection of 2,6-dichloro-4-nitroaniline (DCN) pesticide. The sensing toward nitro-organic pesticides is primarily governed by photoinduced electron transfer instead of FRET. The targeted DCN pesticide contains an electron-withdrawing nitro group, which influences its electronic structure. These groups lower the energy levels of the lowest unoccupied molecular orbitals (LUMOs), making them energetically favorable electron acceptors. DFT calculations further support PET as the dominant mechanism, with pesticides possessing lower LUMO energies and exhibiting greater quenching efficiency. Fluorescence quenching is also influenced by competitive absorption of excitation light (inner filter effect-like behavior). The UV–vis absorption spectra of pesticides coincide with the excitation of the MOF’s wavelength, which lowers the absorption efficiency of the sensor of excitation energy and further reduces fluorescence intensity. The multiple interactions, including π–π stacking interactions, hydrogen bonding, and hydrophobic interactions, contribute to pesticide adsorption. The π–π stacking interactions occur between the benzene rings of the MOF structure and the aromatic rings of DCN. Hydrogen bonding occurs with functional groups containing nitrogen and oxygen. In addition, hydrophobic interactions occur between the MOF framework and the non-polar pesticide molecule. Moreover, the study performed the real sample detection of 2,6-dichloro-4-nitroaniline pesticide. The tomato and apple samples were tested with a naked-eye sensor using a kgd-M1@ACPs probe. Recovery rates of 99.37 to 100.95% and 98.08 to 104.37% were detected for apple and tomato, respectively [60]. Huang et al. synthesized a zeolitic imidazolate framework as an adsorbent, which showed effective removal of nine organochlorine pesticides [61]. Their step-wise synthesis process and adsorption results are described in Figure 5.

### 5.2. Polymer-, MIP-, COF- and Porous Organic Framework-Based Materials

Wang et al. synthesized a dual-functional CD-incorporated MIP (CD@MIP) for the enhancement of MCPA adsorption. According to the Langmuir adsorption model, the maximum adsorption of MCPA was 93.9 mg g^−1^. The adsorption reached equilibrium within 30 min, indicating rapid adsorption. In the range of 10–50 mgL^−1^, the adsorption increases continuously, and saturation occurs at 50 mgL^−1^ of MCPA. In the initial 20 min, the adsorption increases sharply after this equilibrium occurs. This behavior is consistent with monolayer adsorption on a relatively homogeneous set of binding sites. Furthermore, cabbage samples were tested to evaluate the potential of CD@MIP for the removal of MCPA. The prepared CD@MIP exhibits high enrichment ability for MCPA with good recoveries rate of 90.8–95.3%. Thus, CD@MIP shows another example of an integrated sensing and adsorption system. In this system, fluorescent carbon dots provide the selective optical response, and the MIP is used for the recognition and enrichment of MCPA [40].

Qi et al. prepared fluffy ball-like magnetic covalent organic frameworks (COFs) for the adsorption of pyridafenthion, phoxim, pyrimitate, and phorate. The COF composite showed adsorption capacities of 163.9–178.6 mg/g. The adsorption capacity of COF was 10 times greater than the adsorption capacity of the amorphous magnetic composite. The adsorption mechanism was studied using DFT. It was proposed that the adsorption process involves non-covalent interactions like π–π stacking, C–H···π, and C–H···S interactions. The initial adsorption is facilitated by π–π stacking interactions, which occur between the aromatic ring of COF and pesticide molecules. Additionally, C–H···π and C–H···S interactions stabilize the adsorbate–adsorbent complex. Since C–H···S interactions significantly increase the adsorption affinity, the inclusion of sulfur-containing groups in organothiophosphate insecticides is important. The negative interaction energies are calculated and show favorable and spontaneous adsorption, validating these interactions. Overall, high adsorption capacity and effective pesticide removal from aqueous media are made possible by the synergistic contribution of π–π stacking and weak hydrogen-bonding interactions [62].

Ali et al. developed FMMIP for the selective adsorption of malathion and chlorpyri-fos from aqueous systems. Target pesticide molecules can bind selectively due to template-specific recognition sites formed during the imprinting process, which largely control the adsorption mechanism. The adsorption data fit well into the Freundlich isotherm, and both kinetic and isotherm analyses support the predominance of physisorption on heterogeneous binding sites. Electrostatic interactions and hydrogen bonding between functional groups (such as –NH and –OH) of the polymer matrix and the analytes are the primary causes of the interactions between the FMMIP and pesticide molecules. Additionally, it has also been found that adsorption is pH sensitive, with optimum performance observed at pH 7, where functional groups are positively activated for interaction. The adsorption capacity and reusability of FMMIP are attributed to its heterogeneous and multilayer adsorption behavior and reversible binding [39].

Yan et al. reported pyrene-based fluorescent porous organic polymers for the selective detection of trifluralin and dicloran. The sensing mechanism involves photoinduced electron transfer and pesticide–polymer interactions via π–π stacking. Strong interactions are found to occur between the pesticides containing nitro/halogen substituents and the polymer framework. The delocalization of π-electron occurs in the electron-rich polymer. Pesticides with an electron-deficient character (i.e., low LUMO) readily undergo an electron transfer process from the polymer to the pesticides. Significant fluorescence quenching is caused by non-radiative relaxation pathways generated through this charge transfer process. Furthermore, the quenching efficacy is further improved by π–π stacking interactions, which improve the adsorption and relationship between the polymer and analytes. Pesticides can be detected quickly, selectively, and sensitively owing to the synergistic effect of PET and π–π interactions [63].

### 5.3. Carbon-Based Nanomaterials

Adesanmi et al. developed electro-spun carbon nanofibers ( ECNFs) derived from polyacrylonitrile for the efficient removal of OPPs. In this study, authors targeted the ethion pesticide using a graphitized structure of ethion. The π-conjugated domains of ECNFs will facilitate π–π stacking with the aromatic moieties of the organophosphate pesticides.

The adsorption mechanism is primarily governed by π–π interactions, hydrophobic interactions, and van der Waals forces between the carbon nanofiber surface and pesticide molecules. Along with π–π interactions, van der Waals interactions are essential and significant on non-functionalized carbon surfaces. The enhancement in adsorption capacity and efficiency result from the high surface area and porous structure of ECNFs as well as the combined effects of hydrophobic interactions, van der Waals forces, and π–π stacking. Overall, π–π stacking, hydrophobic interactions, van der Waals forces, and the high surface area and porous structure of the ECNFs facilitate the removal of pesticides in an efficient way [64].

### 5.4. Metal Oxide- and LDH-Based Hybrid Materials

Hao et al. synthesized urea-functionalized magnetic layered double hydroxide composite (Urea-Fe_3_O_4_@LDH) for the efficient adsorption of triphenyl phosphate (TPHP). This process is primarily influenced by electrostatic and π–π interactions between the composite and TPHP. The positively charged surface of Urea-Fe_3_O_4_@LDH strongly interacts with the phosphate group of TPHP via the electron-rich oxygen, leading to electrostatic interactions. On the other side, the presence of aromatic benzene rings in TPHP enables π–π interactions with the conjugated structures on the adsorbent surface, enhancing adsorption affinity. The urea functional groups (–NH–CO–NH–) further contribute by providing additional active binding sites and improving surface reactivity. Additionally, the large mesoporous structure of the composite allows efficient diffusion and accessibility of TPHP molecules to active sites. The synergistic effect of electrostatic attraction, π–π interactions, and functional group-assisted binding results in rapid adsorption kinetics and exceptionally high adsorption capacity [65].

**Table 2:** Detection and adsorption performance of representative hybrid nanomaterials for pesticide monitoring and removal.

S No.	Material	Material Category	Pesticide	Detection Function	LOD	LOD Determination Method	Fluorescence Mode	Adsorption Capacity	Adsorption Time	Adsorption Model	Specific Surface Area	Adsorption Mechanism	Average Pore Diameter	Selectivity /Tested Interferents	Ref.
1	Fe_3_O_4_@SiO_2_@UiO-67	MOF-based magnetic hybrid	Glyphosate (GP)	Fluorescence detection + adsorption/removal	LOD = 0.093 mg/L	3Sb/m (Sb = SD of blank; m = calibration slope)	OFF–ON (turn-on)	256.54 mg/g	60 min	Pseudo-second-order model	NR	Chemisorption; Zr–O–P coordination between glyphosate phosphate groups and Zr–OH sites	NR	Other OPPs investigated for detection; selective recognition of glyphosate	[58]
2	Zr-based MOFs (UiO-67)	MOF-based material	Glyphosate (GP), glufosinate (GF)	Adsorption/removal	NR	NR	NR	GP = 537 mg/g; GF = 360 mg/g	GP = 150 min; GF = 200 min	Pseudo-second-order	2172 m^2^/g	Mass transfer/intraparticle diffusion; phosphate–Zr interactions	1.17 and 1.61 nm	NR	[59]
3	Zr-MOFs (DUT-2TFB)	Functionalized MOF	β-Cyfluthrin, λ-cyhalothrin, ethofenprox, bifenthrin	Adsorptive extraction/removal	NR	NR	NR	87.49, 78.99, 80.13 and 81.10 mg/g, respectively	NR	Pseudo-second-order	983.41 m^2^/g	π–π and hydrophobic interactions	NR	Four pyrethroid pesticides investigated	[66]
4	kgd-M1@ACPs	MOF–polymer/alginate hybrid	2,6-Dichloro-4-nitroaniline (DCN)	Fluorescence + naked-eye detection + adsorption/removal	LOD = 0.09 μM	3σ/Ksv (σ = SD, n = 5)	ON–OFF (quenching)	83.3 mg/g	240 min	Pseudo-second-order	NR	π–π stacking, hydrogen bonding and hydrophobic interactions	NR	NIT, NF, PCNB and TMX; DCN showed strongest response	[60]
5	CD@MIP	Carbon dot molecularly imprinted polymer hybrid	2-Methyl-4-chlorophenoxyacetic acid (MCPA)	Fluorescence detection + adsorption/enrichment	LOD = 3.5 μmol/L	3σ method	ON–OFF (quenching)	93.9 mg/g	30 min	Langmuir/pseudo-second-order	16.1 cm^2^/g	Monolayer adsorption; hydrogen bonding and charge-transfer interactions	20.9 nm	Cabbage real samples evaluated; specific fluorescence interferents NR	[40]
6	Magnetic-COF	Magnetic–COF hybrid	Pyridafenthion, phoxim, pyrimitate, phorate	Adsorption/removal	NR	NR	NR	163.9–178.6 mg/g	30 min	Pseudo-second-order	1543 m^2^/g	Hydrogen bonding, electrostatic and π–π interactions	70 nm	Four organophosphorus pesticides investigated	[62]
7	FMMIPs	MIP	Malathion (MLT), chlorpyrifos (CPS)	Adsorption/removal	NR	NR	NR	MLT = 93 mg/g; CPS = 69 mg/g	MLT = 14 min; CPS = 13 min	Freundlich isotherm	NR	Physisorption, electrostatic interaction and hydrogen bonding	250 nm	MLT and CPS used as target analytes; other interferents NR	[39]
8	LNU-45 and LNU-47	Fluorescent porous organic polymers	Chlorothalonil (CTL), nitrofen (NF), trifluralin (TFL), dicloran (DCN)	Fluorescence detection + adsorption	DCN LOD = 0.0155 ppm (LNU-45); 0.0197 ppm (LNU-47)	3σ/Ksv (σ = SD of blank)	ON–OFF (quenching)	NF = 116.0; CTL = 70.0; DCN = 139.8; TFL = 231.9 mg/g	NR	NR	LNU-45 = 322.401 m^2^/g; LNU-47 = 181.924 m^2^/g	Photophysical/electron-transfer interactions; adsorption associated with π-conjugated framework	LNU-45 = 1.165–1.809 nm; LNU-47 = 1.810 nm	NF, CTL, TFL and DCN; strongest quenching for TFL/DCN	[63]
9	E-CNFs	Carbon-based nanomaterial	Ethion	Adsorption/removal	NR	NR	NR	20 mg/g	120 min	Pseudo-second-order	R-ECNFs = 24.46; A-ECNFs = 26.29 m^2^/g	π–π, hydrophobic, and van der Waals interactions	R-ECNFs = 6.78; A-ECNFs = 7.65 nm	NR	[64]
10	Urea-Fe_3_O_4_@LDH	Metal oxide–LDH hybrid	Triphenyl phosphate (TPHP)	Adsorption/removal	NR	NR	NR	589 mg/g	360 min	Pseudo-second-order	58 m^2^/g	Electrostatic and π–π interactions; urea-assisted binding	19 nm	Effects of pH, ionic strength, and humic acid investigated	[65]

Deductively, the reported hybrid materials show pesticide removal in a variety of material classes with numerous interaction mechanisms involving MOF-based coordination, molecular imprinting, π–π interactions, hydrogen bonding, electrostatic interactions, hydrophobic interactions, and diffusion-assisted adsorption. Unfortunately, there are very few multifunctional systems in which the adsorption/removal and detection are achieved using the same material. For instance, Fe_3_O_4_@SiO_2_@UiO-67, CD@MIP, and kgd-M1@ACPs integrate various sensors for the detection of pesticides using fluorescence, which is followed by adsorption or removal. However, differences in influencing factors on the sensing and adsorption processes can lead to differences in the characteristic required for each of these processes, thus limiting the design of innovative systems. For these integrated systems, the main challenge remains ensuring rapid signal generation, high adsorption capacity, selectivity, regeneration, structural stability, and excellent performance in badly contaminated environmental matrices.

## 6. Conclusions and Future Perspectives

Fluorescent inorganic–organic hybrid NPs have attracted considerable attention from researchers for the detection and removal of pesticides. The composite of organic and inorganic materials offers unique physicochemical properties and better interaction with targeted molecules. The main mechanisms in pesticide detection are photophysical processes such as FRET, IFE, PET, and fluorescence quenching. On the other hand, adsorption and photocatalytic degradation contribute to pesticide removal. Pesticide adsorption is highly dependent on interfacial interactions, such as hydrogen bonding, electrostatic attraction, π–π stacking, and coordination. Despite the promising results, there are still several challenges in sensing approaches. The IFE-based approach is relatively simple and cost-effective for detecting pesticides, even in complex matrices, but they are often limited by issues related to sensitivity and interference. For instance, the spectrum overlap and the quencher extinction coefficient have a significant impact on the efficiency of IFE-based systems. When the latter is low, the overall sensing performance may be affected. Furthermore, choosing an appropriate fluorophore–quencher combination is still a crucial aspect that needs to be further optimized. Although some materials are effective in the laboratory setup, their performance decreases in real environmental matrices. There are still several challenges related to selectivity, long-term stability, and reproducibility that need further attention, along with concerns regarding large-scale synthesis and cost.

To advance the field, future designs should incorporate hybrid nanomaterials enabling improved selectivity and long-term resistance stability with enhanced environmental resistance. To address the current challenges, fluorophore–quencher systems incorporating high levels of surface functionalization and specific binding locations should be devised. Developing integrated frameworks for simultaneous pesticide sensing and removal is an attractive pursuit. Among many other considerations, these systems should possess a high level of reusability and be safe to use and deploy in real-world contexts.

## Data Availability

No new data were created or analyzed in this study. Data sharing is not applicable to this article.

## Abbreviations

FRET	Förster resonance energy transfer
IFE	Inner filter effect
PET	Photoinduced electron transfer
H-bonding	Hydrogen bonding
HPLC	High-pressure liquid chromatography
GC-MS	Gas chromatography–mass spectrometry
CDs	Carbon dots
ROS	Reactive oxygen species
PRMD	Pesticide Registration and Management Division
MIP	Molecularly imprinted polymer
SI-ATRP	Surface-initiated atom transfer radical polymerization
ECNFs	Electro-spun carbon nanofibers
CQDs	Carbon quantum dots
LOD	Limit of detection
NR	Not reported
N/A	Not applicable
RF	Ratiometric fluorescence
